# α-Mangostin Inhibits α-Synuclein-Induced Microglial Neuroinflammation and Neurotoxicity

**DOI:** 10.1007/s10571-015-0264-9

**Published:** 2016-03-22

**Authors:** Zhaoyang Hu, Wei Wang, Jing Ling, Chunming Jiang

**Affiliations:** 1 Hangzhou Cancer Institute, Hangzhou Cancer Hospital, Hangzhou, 310007 Zhejiang China; 2 Department of Pharmacy & Medical Appliances, Hangzhou Sanatorium of PLA, No.27 Yang Gong Dike, Hangzhou, 310007 Zhejiang China; 3 Department of Pediatrics, Hangzhou First People’s Hospital, Huansha Road, No.261, Hangzhou, 310003 Zhejiang China

**Keywords:** α-Mangostin, Microglia, α-Synuclein, Neurotoxicity

## Abstract

Microglia-mediated neuroinflammation induced by α-synuclein in the substantianigra likely either initiates or aggravates nigral neuro degeneration in Parkinson’s disease (PD). We aimed to explore the effects of α-mangostin (α-M), a polyphenolicxanthone derivative from mangosteen on α-synuclein-stimulated DA neurodegeneration. Primary microglia, mesencephalic neuron, mesencephalic neuron-glianeuronal cultures, and transwell co-cultures were prepared separately. Liquid scintillation counting was used to determine the radioactivity in DA uptake. Enzyme-linked immunosorbent assay (ELISA) was performed in the IL-1β, IL-6, and TNF-α assay. The expression of proteins was analyzed by Western blot. α-M inhibited the increased levels of pro-inflammatory cytokines, NO, and ROS in α-synuclein-stimulated primary microglia. Mechanistic study revealed that α-M functioned by inhibition of nuclear factor kappa B (NF-κB) and NADPH oxidase. Further, α-M protected α-synuclein-induced microglial and direct neurotoxicity. Although detailed mechanisms remain to be defined, our observations suggest a potential compound, which inhibits microglial activation induced by α-synuclein by targeting NADPH oxidase, might be a therapeutic possibility in preventing PD progression.

## Introduction

Parkinson’s disease (PD), the second most common neurodegenerative disorder only after Alzheimer’s disease (AD), is characterized by the progressive and selective deterioration of dopaminergic neurons in the substantianigra pars compacta (SNpc) (Olanow and Tatton 1999). The exact cause of dopaminergic neuron loss is still obscure. There is a growing body of evidence that suggests uncontrolled neuroinflammation, especially activation of microglia, might play a crucial role in the early onset and progression of PD (Hirsch and Hunot 2009; Lee et al. 2009). Thus, it is widely accepted that development of new inhibitors for pro-inflammatory responses mediated by microglial activation is a potential therapeutic strategy for neuroinflammatory disorders in PD.

Microglia is the resident immune cells in the brain, mainly response to foreign proteins, cell debris, and various adverse molecules. Activation of microglia results in a change in cell morphology from a resting ramified shape to an amoeboid profile, accompanied by continued release of inflammatory neurotoxic products. For example, inflammatory cytokines including interleukin (IL)-1β, IL-6, and tumor necrosis factor-α (TNF-α) and additionally, reactive oxygen species (ROS) and inducible nitric oxide synthase (iNOS) were increased in cerebrospinal fluid of PD patients, which could further impact on neurons to induce progressive neurodegeneration (Montgomery and Bowers 2012; Blum-Degen et al. 1995). Now, a number of pro-inflammatory stimuli that mediate microglial activation have been well illustrated in vitro and in vivo (Perry et al. 2010; Tansey and Goldberg 2010). Among these, classical agent lipopolysaccharide (LPS) was used to establish the PD models by infusion of LPS into SN pars compacta and LPS could directly activate microglia. As for other neurotoxins, α-synuclein, the major component of Lewy bodies can cause neurodegeneration in the aggregated form and play a prominent role in microglial activation by enhanced production of pro-inflammatory cytokines, thereby resulting in persistent and progressive nigral neurodegeneration in PD (Sanchez-Guajardo et al. 2013; Zhang et al. 2005). As a result, regulating the activated microglia induced by α-synuclein might be an important strategy for the treatment of neurodegenerative disorder.

Natural products from traditional medicinal plants, especially which therapeutic applications and benefits have been documented by traditional medicine systems, have been identified as an important potential resource for discovery of lead compounds for multiple diseases (Campos et al. 2011). α-Mangostin (α-M), a polyphenolicxanthone extracted from the pericarp, bark, or dried sap of mangosteen (*Garciniamangostana* Linn), has a long medical history in tropic countries. Due to its broad range of bioactivities, α-M has been used to cure for abdominal pain, diarrhea, dysentery, infected wound suppuration, or chronic ulcer according to traditional medicine documents (Pedraza-Chaverri et al. 2008). Recent studies have proved that α-M, acting as a powerful ROS scavenger, protects neurons from mitochondrial toxin 3-nitropropionic acid-induced cell death (Pedraza-Chaverri et al. 2009). Moreover, it is also shown to have remarked anti-inflammatory activity as well as anti-cancerogenic activity in macrophage cells (Tewtrakul et al. 2009). Considered that α-M is a restricted plant polyphenol xanthone that exerts strong anti-inflammation and anti-oxidative activity, α-M might affect the microglial activation and possess neuroprotective activity.

In this paper, we aim to evaluate the beneficial effects of α-M on α-synuclein-induced microglial activation and α-synuclein-mediated and direct neurotoxicity. Our results showed that α-M inhibited the increased productions of pro-inflammatory cytokines and NO in α-synuclein-stimulated primary microglia cells. Furthermore, α-M protected α-synuclein-induced direct neuronal cell death and inhibited microglial neurotoxicity. Mechanistic study revealed that α-M suppressed α-synuclein-induced inflammation via the inhibition of nuclear factor kappa B (NF-κB) activation and reduced ROS production by blockade of NADPH oxidase. Together, our data indicate α-M inhibits α-synuclein-induced microglial neuroinflammation and neurotoxicity.

## Materials and Methods

### Chemicals

The purified human α-synuclein was obtained from Biomart (Shanghai, China). Cell culture reagents were purchased from Gibco (Grand Island, NY). Antibodies against IκB-α, p65, p-p65, MAP2, and β-actin were acquired from Cell Signaling Technology (Beverly, MA). IBA-1 was obtained from Abcam (Cambridge, MA). Griess reagents were obtained from Beyotime (Jiangsu, China). IL-1β, IL-6, and TNF-α ELISA kits used in this study were obtained from Maibio (Shanghai, China). CCK-8 reagent was from Dojindo (Tokyo, Japan). α-Mangostin was bought from Sigma-Aldrich (USA).All compounds otherwise indicated were also purchased from Sigma (St. Louis, MO).

### Primary Cultures

Primary microglia cultures were prepared according to the “shaking off” method as described previously (Maezawa et al. 2006). All animals were used in accordance with the guidelines of Hangzhou First People’s Hospital, the animal experiment ethics committee approval No. ZJDL [2013] 31. After removing the meninges from newborn 24-h wild-type SD rats, the brain was dissected, minced in cold Dulbecco’s modified eagle medium (DMEM), then centrifuged and re-suspended in DMEM supplemented with 10 % fetal bovine serum (FBS). After 14 days, microglia suspensions were collected by shaking the flasks on a shaker (65 rpm, 4–6 h, 37 °C), and then seeded in DMEM with 10 % FBS. The purity of cultures was ≥99 % for microglia as identified by anti-IBA-1 immunostaining. Primary microglia cultures were used to determine the effects of α-Mangostin on microglial activation.

To investigate the effects of microglia-mediated neurotoxicity, conditioned medium (CM) was collected from microglia for treating midbrain neuron-enriched cultures. The prepared primary microglia were cultured in 12-well culture plates at a density of 1.0 × 10^6^ cells/well for 24 h in DMEM with 5 % FBS. Cultures were washed twice by PBS and added the Neurobasal medium without serum for another 24 h. The NB/B27-based microglia CMs were collected, centrifuged, and used immediately for future uses.

Mesencephalic neuronal cultures were prepared from newborn SD rats according to the previous method (Domico et al. 2006). In short, the mesencephalon was dissected, triturated to dissociate the cells, and filtered through a 30-μm Nytex filter before counting in a hemocytometer. Cells were plated at 2.0 × 10^5^ cells/cm2 on polyornithine- and serum-coated wells. Cultures were kept at 37 °C in a 95 % air/5 % CO_2_ atmosphere with 100 % relative humidity. Culture media were renewed with 1 ml of fresh DMEM 24 h after plating. 5-Fluoro-2-deoxyuridine (50 μM) plus uridine (10 μM) was present from days 7–9 in vitro to reduce glial growth. Cells were supplemented with 5.5 mM glucose approximately every third day until the conclusion of the study. Mesencephalic neuronal cultures were used to determine the effects of α-Mangostin on microglial neurotoxicity.

### Mesencephalic Neuron-Glia Cultures

Neuron-glia cultures were prepared from the ventral mesencephalic tissues of newborn 24-h wild-type SD rats as described previously (Qin et al. 2002). In brief, dissociated cells were seeded at a density of 5 × 10^5^/well in poly-d-lysine-coated 24-well plates. After 7 days, mesencephalic neuron-glia cultures were used for treatment, which contained about 10 % microglia, 40 % astrocyte, and 50 % neurons. Mesencephalic neuron-glia cultures were used for the DA uptake assay to investigate the effect of α-Mangostin on microglial neurotoxicity.

### DA Uptake Assay

After indicated treatment, cells were incubated for 20 min at 37 °C with 1 mM ^[3H]^DA (PerkinElmer Life Sciences, Boston, MA, USA) in Krebs–Ringer buffer (16 mM sodium phosphate, 119 mM NaCl, 4.7 mM KCl, 1.8 mM CaCl_2_, 1.2 mM MgSO_4_, 1.3 mM EDTA and 5.6 mM glucose). After washing with ice-cold Krebs–Ringer buffer for three times, cells were collected and dissolved in NaOH. Radioactivity was determined by liquid scintillation counting. Nonspecific dopaminergic (DA) uptake determined in the presence of mazindol (10 mM) was subtracted from total uptake to obtain specific DA uptake.

### ELISA

The culture medium was collected after treated with 200 nM α-synuclein or various concentrations of α-synuclein. After centrifuged at 2500 rpm for 5 min, the supernatant was stored at −20 °C before used. The levels of pro-inflammatory cytokines, including IL-1β, IL-6, and TNF-α, were determined by sandwich ELISA assay according to the manufacturer’s instruction.

### Griess Reaction

The concentration of nitrite (NO_2_^−^) due to enhanced expression of iNOS in activated microglia was measured by a colorimetric method based on Griess reagent. The optical density (OD) was determined at 550 nm by Varioskan Flash plate reader (Thermo Scientific, PA, USA).

### Immunofluorescence Staining

Microglial cells or neurons were fixed in 4 % paraformaldehyde for 1 h and blocked in 10 % BSA for 1 h at room temperature. Microglial cells were stained with rabbit anti-Iba1 antibody, and neurons were immunostained with anti-MAP2 antibody overnight at 4 °C. After incubation with anti-rabbit IgG antibody, cells were counterstained with DAPI. Cells were then imaged by using a Leica TCS SP2 AOBS confocal microscope (Leica Microsystems, GmbH, Wetzlar, Germany) with a 20 × objective.

### Western Blot

After incubation with α-synuclein and α-M, cells were washed twice with ice-cold PBS, harvested by scraping with RIPA lysis buffer, and quantified with BCA. Lysates were separated by SDS-PAGE and transferred onto polyvinylidenedifluoride (PVDF) membranes. Membranes were blocked for 1 h with 5 % milk/TBST and incubated with primary antibodies above overnight at 4 °C. The membranes were then incubated with a horseradish peroxidase-conjugated anti-rabbit antibody for 1 h before using Enhanced Chemoluminescence Plus (Thermo, Pittsburgh, PA). Image acquisition was completed by LICOR Odyssey Infrared Imaging System (Lincoln, NE), and analysis of band densities was performed in Image J 5.0.

### Transwell Co-cultures

Transwell co-cultures were performed as previously described (Lee et al. 2010). Microglia were plated onto the top side of the transwell inserts (0.4 μm pore size polyester membrane pre-coated with poly-l-lysine) at the cell density of 2.0 × 10^5^ cells/cm^2^. The transwells were positioned approximately 2 mm above the neuron-enriched culture plate, and the microglia grown on the transwells were separated from the neurons by the permeable transwell membrane. Then, α-synuclein (200 nM), α-M (100 nM), α-synuclein plus α-M or DMSO as a solvent control were added to the media below the transwells. Transwell co-cultures were used to directly observe the interaction between α-M-induced inhibition of microglial activation and neuroprotection.

### Cytotoxicity Assay

Cell viability was evaluated by the 3-(4,5-dimethylthiazol-2-yl)-2,5-diphenyltetrazolium bromide (MTT) reduction assay. In brief, neurons (5 × 105 cells/mL) and microglia (3 × 105 cells/mL) were seeded in the transwell system, treated as described above. After 24 h of incubation, the medium was removed. The neurons and microglia were separated and then incubated with 0.5 mg/mL MTT solution. After incubation for 3 hours at 37 °C in 5 % CO2, the supernatant was removed, and the formation of formazan crystals was measured at 490 nm with a microplate reader.

### Statistical Analysis

The results of all experiments were expressed as mean ± standard error of the mean (SEM), and data were analyzed by analysis of variances (ANOVA) followed by Dunnett’s test using GraphPad Prism 5.0 statistical analysis software, with p-values less than 0.05 considered statistically significant.

## Results

### Effect of α-M on Pro-inflammatory Cytokines Production in α-Synuclein-Stimulated Microglia

**Fig. 1:**
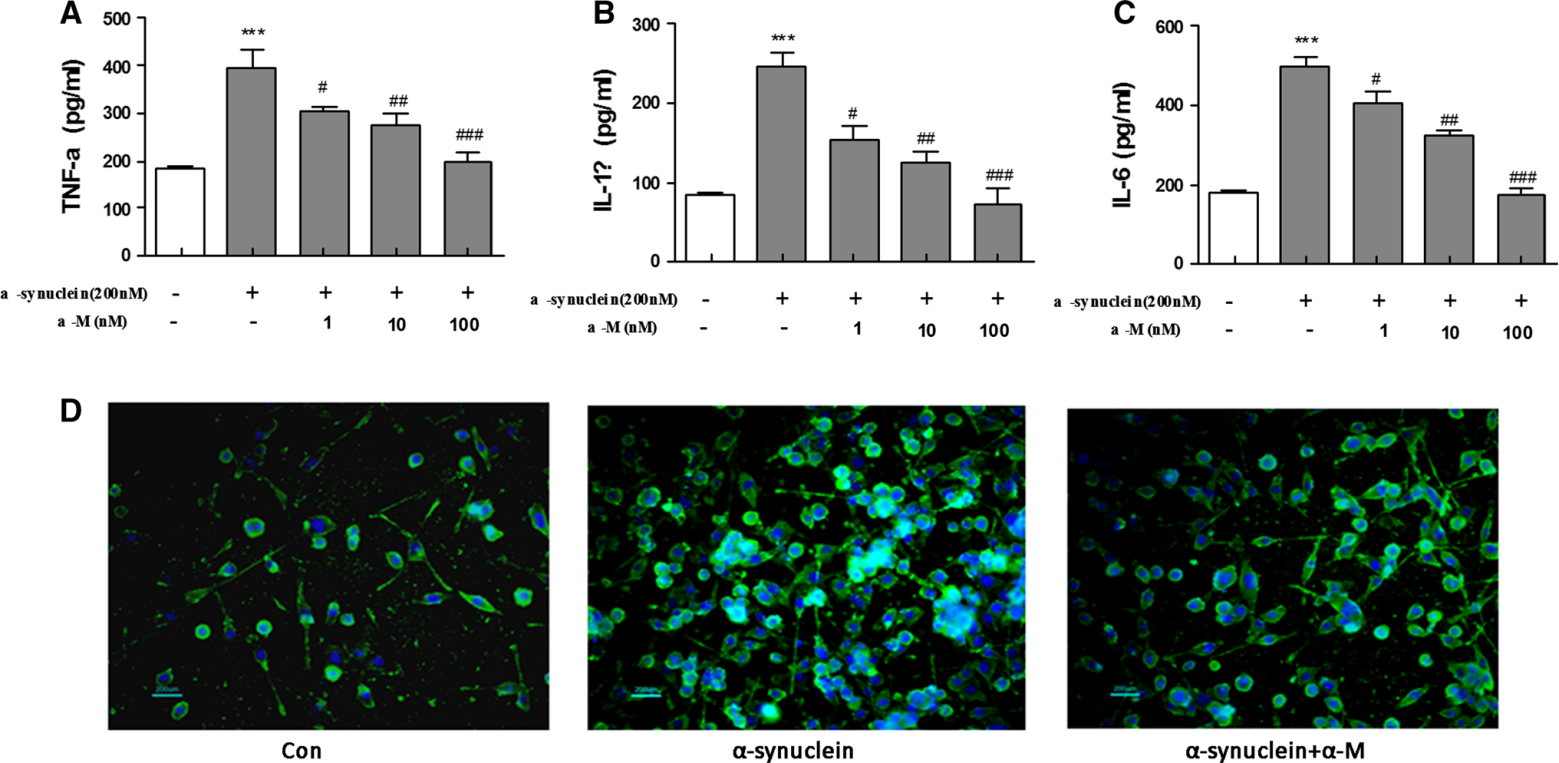
Effect of α-M on α-synuclein-induced production of proinflammatory cytokines and microglial activation. The cytokines production of TNF-α (**a**), IL-1β (**b**) and IL-6 (**c**) in the media were assayed by ELISA. The morphology of microglia was determined by IBA-1 immunofluorescence (**d**). The results are expressed as mean ± S.E.M for three independent experiments. ***p < 0.001 versus control, ^ #^ *p* < 0.05 versus α-synuclein, ^ ##^ *p* < 0.01 versus α-synuclein and ^ ###^ *p* < 0.001 versus α-synuclein. * Scale bar* = 200 μm

Pro-inflammatory cytokines, such as IL-1β, IL-6, and TNF-α, play crucial roles in microglia-mediated neuroinflammation in neurodegenerative disorder. To investigate the effects of α-M on α-synuclein-induced pro-inflammatory cytokines production, aging of commercially purified human α-synuclein (200 nM) was used to activate primary rat microglial cells, followed by treatment with α-M in a concentration range of 1, 10, 100 nM for 24 h. Consistent with previous reports, α-synuclein resulted in an increase of the pro-inflammatory cytokines production, including IL-1β, IL-6, and TNF-α (Forstermann et al. 2012). Subsequently, we found that treatment with α-M significantly inhibited pro-inflammatory cytokine productions in a dose-dependent manner (Fig. 1a–c), suggesting that α-M could reverse the α-synuclein-induced pro-inflammatory cytokines production in primary microglia. Traditionally, the microglial cells were classified into two primary phenotypic states in vivo: quiescent or activated. Then, the identity of the activated cells was confirmed by their immunoreactivity to anti-ionized calcium binding adaptor molecule 1 (IBA-1), a microglia-specific marker. A distinct activation phenotype of microglia is shown in Fig. 1d by IBA-1 after 200 nM α-synuclein treatment for 24 h. Moreover, the morphology changes induced by α-synuclein were restored by treatment of 100 nM α-M for 24 h, indicating the inhibiting effect of α-M on microglial activation.

### Effect of α-M on NO Production and iNOS Expression in α-Synuclein-Stimulated Microglia

**Fig. 2:**
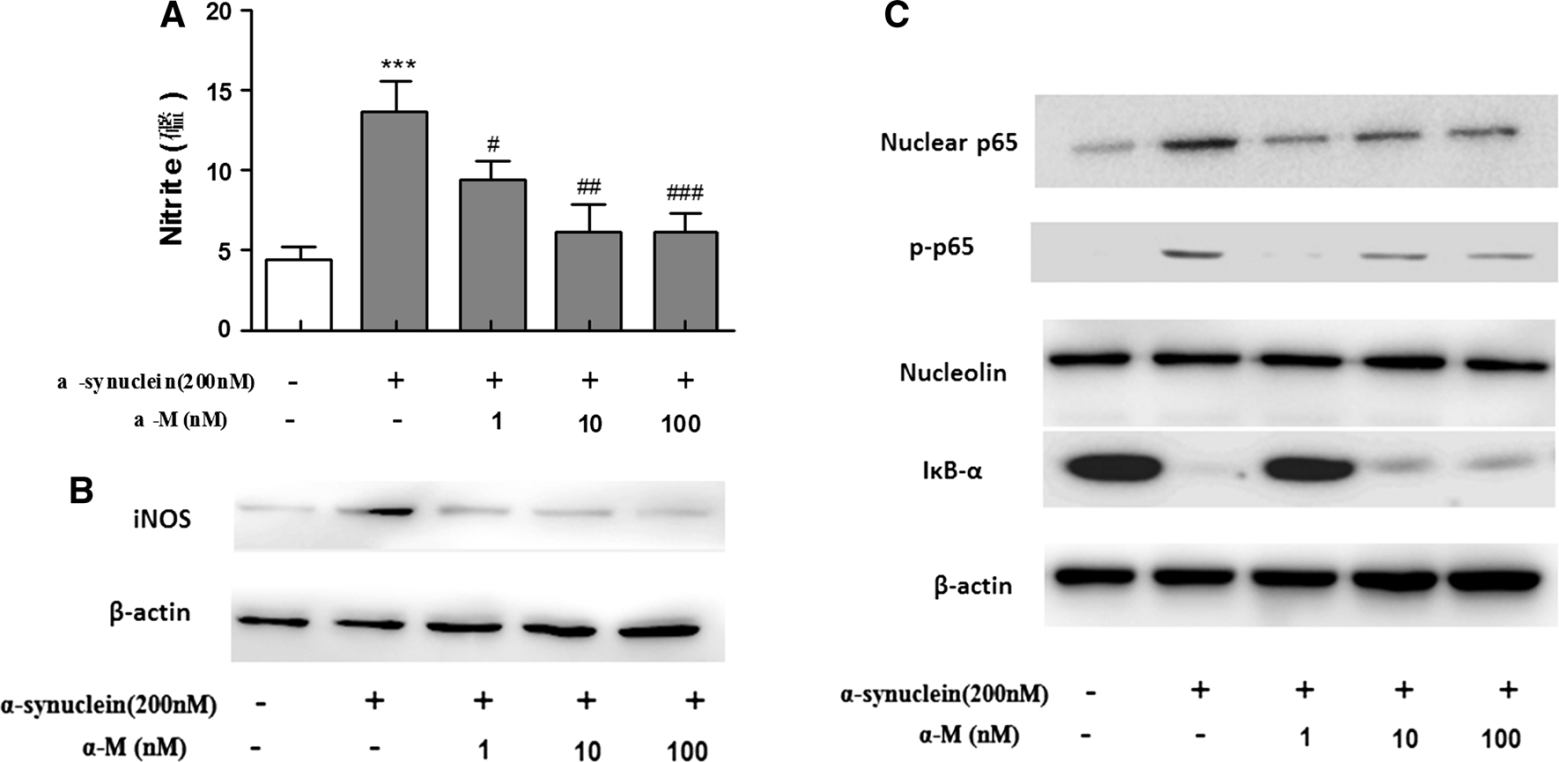
Effect of α-M on α-synuclein-induced production of nitrite production and iNOS expression. ** a** The nitrite in the medium was determined using Griess reagent. ** b** The protein expression level of iNOS was determined by western blot analysis. ** c** Effects of α-M on NF-κB pathway in α-synuclein-stimulated microglia. Nucleolin and β-actin were used as internal controls. ***p < 0.001 versus control, ^ #^ *p* < 0.05 versus α-synuclein, ^ ##^ *p* < 0.01 versus α-synuclein and ^ ###^ *p* < 0.001 versus α-synuclein

NO is also an important mediator of inflammation in central nervous system, which is synthesized by iNOS (Baker et al. 2011). To investigate the effect of α-M on α-synuclein-stimulated NO production, we measured nitrite accumulation by using Griess reagent and iNOS expression by Western blot in primary rat microglial cells. Stimulation of primary microglial cells with 200 nM α-synuclein markedly increased the nitrite production and iNOS expression compared to the normal cells. However, treatment with α-M at indicated concentrations significantly suppressed the α-synuclein-increased nitrite production in a dose-dependent manner (Fig. 2a) and consistently reduced the iNOS expression in primary microglial cells (Fig. 2b).

### α-M Inhibits NF-κB Activation Induced by α-Synuclein in Primary Microglia

It is widely known that NF-κB signaling pathways are key transcriptional regulators of pro-inflammatory cytokines and iNOS in activated stimulated microglial cells (Choi et al. 2012). To investigate whether α-M inhibited the production of pro-inflammatory cytokines and NO by blockade of NF-κB pathway, we next examined the effect of α-M on the activation of NF-κB signaling pathways, namely, the degradation of IκB-α in the cytosol, phosphorylation level, and nuclear translocation of p65 in α-synuclein-stimulated primary microglial cells. As shown in Fig. 2c, α-M attenuated α-synuclein-enhanced degradation of IκB-α in cytosol in a concentration-dependent manner (Fig. 2c). Also, α-M reduced the level of α-synuclein-induced phosphorylated p65 subunit and the translocation of NF-κB p65 subunit into nucleus from cytoplasm (Fig. 2c). Here, we demonstrated that inhibition of NO production and pro-inflammatory cytokines by α-M might also be linked through the blocking of NF-κB signaling pathway that inactivated primary microglia.

### Effect of α-M on ROS Production in α-Synuclein-Activated Microglia

**Fig. 3:**
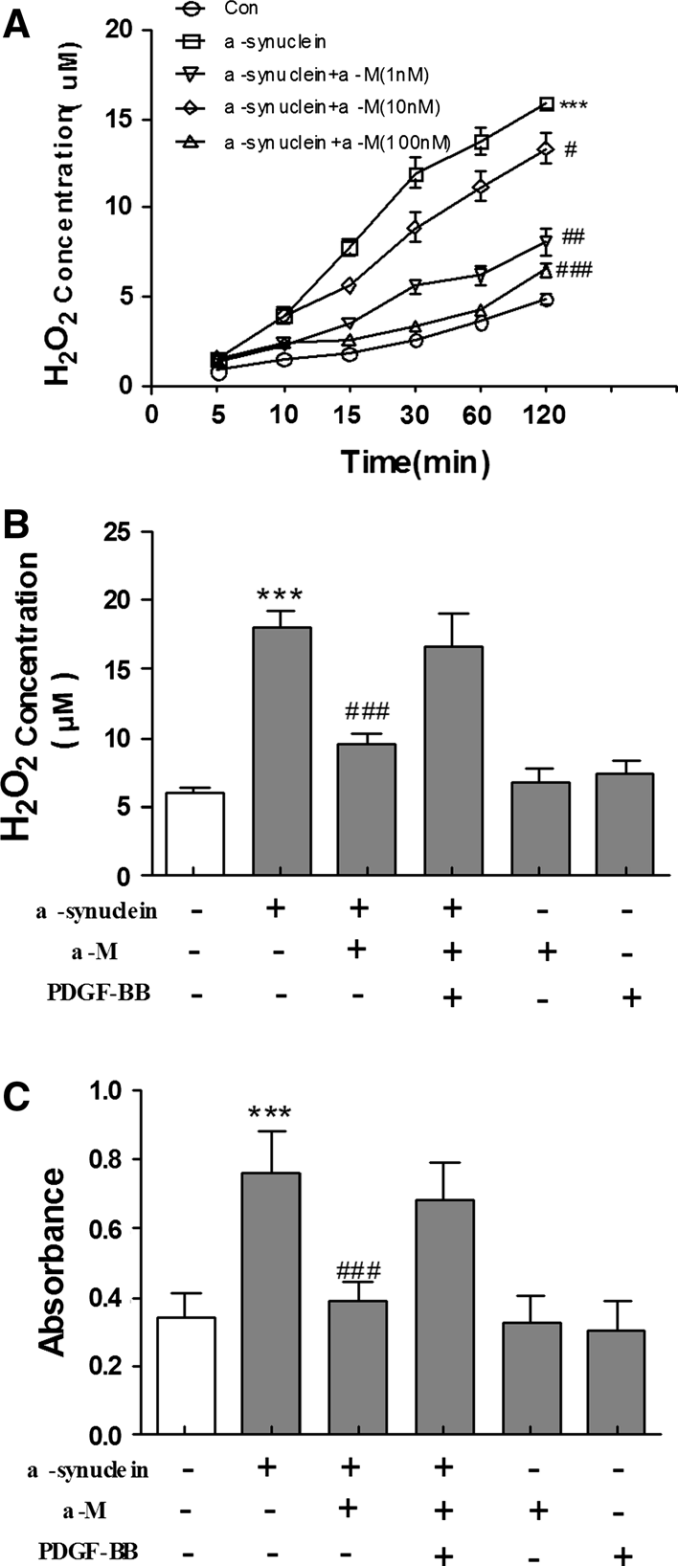
Effects of α-M on extracellular H_2_O_2_ release in α-synuclein-stimulated microglia. ** a** Microglial cells were stimulated by α-synuclein with or without α-M. **b**, ** c** After pretreat with NOX-1-pcDNA3.0 for 24 h, H_2_O_2_ concentrations in the medium were measured at the indicated points. The concentration of H_2_O_2_ and superoxide in cultural supernatants were measured by Amplex Red or Wst-1, respectively. ***p < 0.001 versus control, ^ #^ *p* < 0.05 versus α-synuclein, ^ ##^ *p* < 0.01 versus α-synuclein and ^ ###^ *p* < 0.001 versus α-synuclein

Some studies suggest that aggregated α-synuclein plays an important role in neurotoxicity by increasing the formation of ROS such as hydrogen peroxide (H_2_O_2_), which can further regulate microglial inflammatory response to a variety of stimuli (Baker et al. 2011). We subsequently quantitatively analyzed the effects of α-Mon H_2_O_2_ release induced by α-synuclein using Amplex Red reagent. As shown in Fig. 3a, exposure to α-synuclein resulted in a marked accumulation of H_2_O_2_ in the culture medium of primary microglial cells (*p* < 0.001 compared to medium treatment). The extracellular H_2_O_2_ concentration reached a near-maximum level at 1 h after 200 nM α-synuclein stimulation but was significantly reduced by α-M treatment in a concentration-dependent manner (Fig. 3a).

NADPH oxidase is a series of enzymes that are involved in producing superoxide and is highly expressed in innate immune cells including microglia (Zhao et al. 2011). Innate immune gene induction in brain involves positive loops of pro-inflammatory gene induction that converge upon the transcription factor NF-κB that is activated by ROS (Phani et al. 2012). To further determine the role of NADPH oxidase in α-M-induced inhibition of microglial activation, we examined whether overexpression of NADPH oxidase-1 could attenuate the protective effects of α-M treatment. However, overexpression of NADPH oxidase-1 by transfection of pcDNA3.0 had inversely increased α-M-downregulated H_2_O_2_ release (Fig. 3b). Next, we detected the concentrations of superoxide in the medium of cell cultures, and proved that α-synuclein effectively evoked superoxide production. Similar results were found in superoxide production, namely, α-M-downregulated superoxide production was decreased by overexpression of NADPH oxidase-1 (Fig. 3c). These results strongly indicate that NOX-1 is likely to be the source of observed H_2_O_2_ release upon α-synuclein stimulation and α-M inhibited α-synuclein-induced ROS production by targeting NOX.

### α-M Protects DA Neurons from α-Synuclein-Induced Microglial and Direct Neurotoxicity

**Fig. 4:**
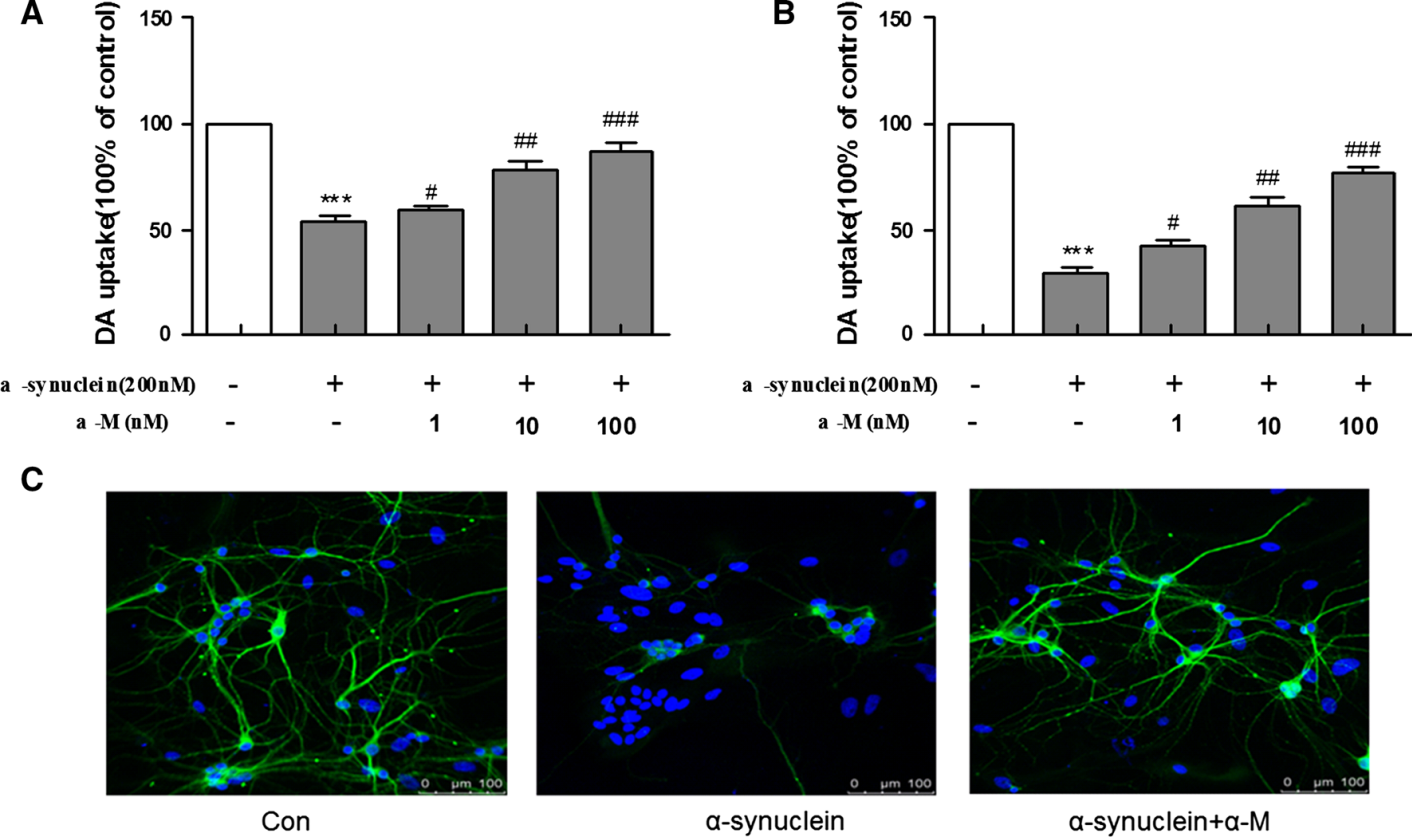
Neuroprotective effects of α-M on α-synuclein-stimulated DA neurodegeneration in rat primary mesencephalic neuron-glia cultures. ** a** Conditioned medium from microglia stimulated with 200 nM α-synuclein caused microglial neurotoxicity measured by [3H]DA uptake. ** b** The mesencephalic neuron-glia cultures seeded in 24-well culture plates were treated with 200 nM α-synuclein and indicated doses of 100 nM α-M. ** c** For visualization of individual dendrites, dendrites of the sparsely plated neurons were demonstrated by immunostaining for a dendritic marker MAP2 (*green*). ***p < 0.001 versus control, ^ #^ *p* < 0.05 versus α-synuclein, ^ ##^ *p* < 0.01 versus α-synuclein and ^ ###^ *p* < 0.001 versus α-synuclein. * Scale bar* = 100 μm (Color figure online)

To investigate the potential values of α-M for treating neurodegenerative disorders, we first tested the protective effects of α-M on midbrain neuron-enriched cultures. Primary microglia were activated by using 200 nM α-synuclein for 24 h and transferred the microglia-conditioned medium (CM) to midbrain neuron-enriched cultures. In our studies, we treated neurons with CMs for 7 days, which were derived from microglia mock-treated with solvent (Con-CM) or α-synuclein (α-s-CM) for 24 h, followed by treatment with or without α-M for 24 h. The assay of DA uptake showed that α-synuclein treatment reduced the uptake capacity of DA by 55 %, and this reduction was dramatically blocked by α-M treatment in a dose-dependent manner (Fig. 4a), suggesting that the addition of α-M to the medium did inhibit the α-synuclein-induced microglial neurotoxicity.

Subsequently, we investigated whether α-M could also protect against direct neurotoxicity induced by α-synuclein, in the rat primary mesencephalic neuron-glia cultures. ^[3H]^DA uptake assay was used to assess the viability of DA neurons in rat primary mesencephalic neuron-glia cultures in which approximately 1 % of the neurons are DA. Various concentrations of α-M (1–100 nM) were added 7 days after the initial seeding, and the viability of DA neurons was assessed by DA uptake assays 7 days after α-M addition. Results indicated that α-M induced survival-promoting effects in a dose-dependent manner and significantly protected DA neurons from spontaneous neuronal death (Fig. 4a). To assess the role of microglia in the α-M-induced survival-promoting effects in this in vitro system, microglia-depleted rat primary mesencephalic neuron-glia cultures were treated with α-M, and DA uptake assays were performed 7 days after α-M treatment (Fig. 4b). These results indicate that microglia play a negligible role in α-M-induced survival-promoting effects.

To further confirm the direct neuronal damage induced by α-synuclein, we found that mesencephalic neurons treated with α-synuclein showed much more robust signs of dendritic damage compared to normal cells. Importantly, 100 nM α-M treatment has protected neurons against α-synuclein stimulation, as demonstrated by immunofluorescent stains with microtubule-associated protein 2 (MAP2), a dendritic marker (Fig. 4c). Therefore, we confirmed the protective role of α-M in α-synuclein-induced microglial and direct neurotoxicity of DA neurons.

### α-M Exerts Neuroprotection by Inhibition of α-Synuclein-Induced Microglial Activation

To directly observe the interaction between α-M-induced inhibition of microglial activation and neuroprotection, we used a transwell co-culture system including primary neurons and microglia and stimulated the cells with α-synuclein (200 nM). Microglia were plated onto the transparent polyester membrane of the transwell inserts, and neurons were placed on the wells below the polyester membrane; as a result, the microglia grown on the transwells were separated from the neuron-enriched cultures by the permeable transwell membrane.

**Fig. 5:**
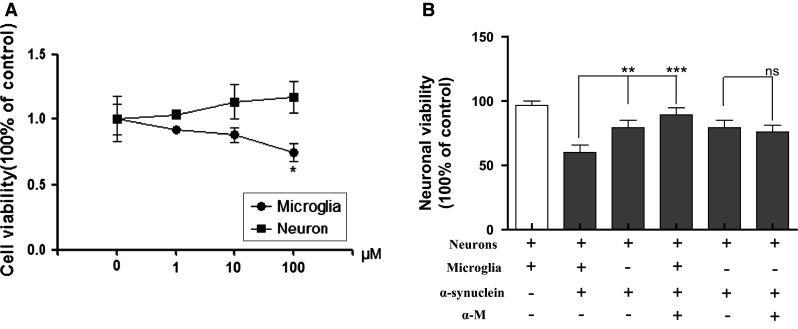
Effects of α-M on neuronal viability in the transwell co-culture of neurons and microglia. ** a** Cell viability following the administration of various concentrations of α-M. ** b** The neuronal viability in co-culture and single-culture systems. ** *p* < 0.01 versus indicated group, *** *p* < 0.001 versus indicated group and ns * p* > 0.05 versus indicated group

To determine the optimal concentration of α-M for cell co-culture, α-M (1, 10, and 100 nM) was applied separately. We found that 100 nM α-M has reduced microglial viability compared with the control (*p* < 0.05), whereas the cell viability in the 1 and 10 nM α-M treatment groups did not significantly differ from that in the control group (*p* > 0.05, Fig. 5a). However, α-M (1, 10 and 100 nM) slightly increased the neuronal viability when compared with the no-curcumin control (*p* < 0.05, Fig. 5a). Accordingly, 10 nM was chosen as the optimal concentration for the transwell co-culture system.

As shown in Fig. 5b, α-synuclein (200 nM) significantly reduced the cell viability of neurons, whereas neuronal viability in co-cultured neurons was significantly lower than that in the single-culture group (*p* < 0.01), indicating that microglial activation played an crucial role in the nerve damage stimulated by α-synuclein. Next we examined the neuronal protection of α-M under various conditions. In the co-culture groups, the administration of α-M after α-synuclein stimulation significantly reversed the down-regulation of neuronal viability (*p* < 0.001). In contrast, in the single-culture groups, α-M after α-synuclein stimulation did not significantly increase the down-regulation of neuronal viability (*p* > 0.05, ns, Fig. 5b).

## Discussion

Increasing evidence has showed that inflammation in the central nervous system is closely associated with the pathogenesis of neurodegenerative disorders, including PD (Zhang et al. 2011). The activation of glial cells, especially microglia, is identified as the hallmark of neuroinflammation because pro-inflammatory cytokines and free radicals are produced by microglia in response to neuronal injury or immunological challenges. Based on the possibility that microglial activation may initiate a cascade of events leading to progressive neurodegeneration, and functions as a major factor to trigger the onset of the degeneration of nigral dopaminergic neurons, to inhibit the microglial activation, related dopaminergic neurodegeneration has become an attractive strategy in PD treatment (Ulusoy et al. 2012).

It has been increasingly known that α-synuclein, the major component of the Lewy body, is a crucial molecular contributor to PD pathogenesis because it is co-localized with the vulnerable dopaminergic neurons; abnormal expression or conformation of α-synuclein may result in early neurological damage in PD (Auluck et al. 2010). The mechanism of α-synuclein-mediated pathogenesis has been suggested to be closely related with microglial activation in human PD brains. Consistent with previous reports (Beraud et al. 2013), we have confirmed that α-synuclein-activated primary microglia, including morphological changes, enhanced production of pro-inflammatory cytokines, NO, and ROS. Therefore, inhibition of α-synuclein-mediated microglial activation and neurotoxicity is identified as a potential therapeutic strategy for PD.

NO is an important biological molecular that mediates the neurotransmission, neurotoxicity, and vasodilation. Excessive generation of NO induced by α-synuclein is rather harmful to neurons in the inflammation-mediated neurodegenerative process (Smith et al. 2012). Increased pro-inflammatory cytokines production stimulated by α-synuclein has been shown to be involved in the development of inflammation in CNS through the destruction of blood–brain barrier, induction of adhesion molecules, and release of chemokines, which facilitates the infiltration of leukocytes into the brain (Imaizumi et al. 2012). In addition, α-synuclein directly induces mitochondrial dysfunction and increases oxidative stress (Cristovao et al. 2012). In the present investigation, we found that α-M, a polyphenolicxanthone derivative from mangosteen, inhibited the α-synuclein-stimulated pro-inflammatory cytokines production, NO release, and ROS in primary microglial cells.

Now, activation of NADPH oxidase is realized as one of the major steps in α-synuclein-stimulated oxidative bursts, which is closely associated with α-synuclein-induced neurotoxicity (Kim et al. 2000). A study by Wei Z et al. has showed that knocking out NADPH oxidase strongly protected dopaminergic neurotoxicity regardless of the dosage of α-synuclein, further indicating that generation of ROS by activated NADPH oxidase is a critical step in α-synuclein-mediated neurotoxicity (Zhang et al. 2005). Importantly, our results found that antioxidant α-M could reduce α-synuclein-induced ROS production by blockade of NOX-1.

Although particular mechanisms remain unclear, α-synuclein-induced neurotoxicity seems to be associated with higher density of microglia in the SN. The enhancement of α-synuclein-induced dopaminergic neurotoxicity by activated microglia signifies an important mechanism underlying PD’s pathological process. Consistently, in our studies, α-synuclein was found to activate primary microglia, which in turn further enhanced α-synuclein-mediated neurotoxicity. Moreover, using different primary mesencephalic cultures, we have demonstrated that α-M was found to interfere with the pathogenesis of microglial activation by inhibition of NADPH oxidase, therefore inhibiting microglial or direct neurotoxicity caused by α-synuclein. Here, our observations indicate a potential compound α-M, which inhibits microglial activation induced by α-synuclein by targeting NADPH oxidase, might be a therapeutic possibility in preventing PD progression.

## Abbreviations

PD: Parkinson’s disease
AD: Alzheimer’s disease
α-M: α-Mangostin
NF-κB: Nuclear factor kappa B
SNpc: Substantianigra pars compacta
ROS: Reactive oxygen species
PVDF: Polyvinylidenedifluoride
